# SET-NUP214-induced hypermethylation landscape promotes abnormal overexpression of HOXC cluster genes in acute megakaryoblastic leukemia

**DOI:** 10.1016/j.gendis.2024.101320

**Published:** 2024-05-07

**Authors:** Akhilesh Kaushal, Samrat Roy Choudhury

**Affiliations:** Pediatric Hematology-Oncology, Department of Pediatrics, Arkansas Children's Research Institute, University of Arkansas for Medical Sciences, Little Rock, AR 72202, USA

The SET-NUP214 gene fusion is mainly found in patients primarily diagnosed with T-cell acute lymphoblastic leukemia (T-ALL) and rarely in acute myeloid leukemia (AML).^1^ Patients with the SET-NUP214 fusion protein (referred to as SN-214) have poor responses to allogeneic stem cell transplantation and a three-year disease-free survival rate of less than 40%.^2^ At the molecular level, the SN-214 fusion is known to recruit chromosomal region maintenance 1 (CRM1) and disruptor of telomeric silencing 1-like (DOT1L) proteins to the promoters of homeobox (*HOX*)-cluster genes, which is consistent with the overexpression of these genes in SN-214^+^ T-ALL.^1^ In contrast, silencing of the tumor suppressor gene, TET methylcytosine dioxygenase 2 (*TET2*), has been observed in the SN-214^+^ T-ALL cell line LOUCY due to hypermethylation at the promoter.^3^ However, the mechanism by which SN-214 alters the DNA methylation (DNAm) landscape to regulate leukemic transcriptional programs and signaling cascades, particularly in the myeloid lineage of leukemia, remains unexplored. Due to the rarity of the disease and the unavailability of genomic data from primary AML samples, our study focused on the MEGAL (ACC719, DSMZ) cell line. This cell line was identified as the only AML cell line expressing SN-214, and we used it to investigate the role of DNAm in leukemic transcriptional programs. To capture genome-wide DNAm and expression, we used the Infinium MethylationEPIC v2.0 BeadChip array and RNA sequencing, respectively. Considering the megakaryoblastic (M7, FAB classification) lineage of the MEGAL cell line, we analyzed the epigenetic changes in comparison to CD41^+^ megakaryocyte (MK) progenitors differentiated from umbilical cord blood-derived CD34^+^ hematopoietic stem progenitor cells (HSPC). We also analyzed the chromatin immunoprecipitation-sequencing data from the LOUCY cell line to identify possible overlaps of the transcription factor CTCF and chromatin marks (H3K4me1, H3K4me3, H3K27ac, H3K36me3, and H3K27me3) with differentially methylated CpGs (mC) in MEGAL. This integrative epigenetic analysis helped us understand the impact of the SN-214 fusion on leukemic transcriptional programs.

After confirming the expression of SN-214 fusion in MEGAL cells (Fig. 1A), we conducted transcriptome profiling that revealed that 44% (*n* = 3820) of genes were up-regulated in MEGAL compared with HSPC, and 46% (*n* = 3491) of genes were up-regulated compared with MK. Similarly, 55% (*n* = 4839) of genes were down-regulated in MEGAL compared with HSPC, and 53% (*n* = 4050) were down-regulated compared with MK (Table S1, 2). We observed 18 *HOX*-cluster genes, including *HOXA10, HOXA11*, *HOXA13*, *HOXA2*, *HOXA7*, *HOXB8*, *HOXB9*, *HOXC4*, *HOXC6*, *HOXC8*, *HOXC9*, *HOXC10*, *HOXC12*, *HOXC-13*, *HOXC13-AS*, *HOXC13-AS1*, *HOXC13-AS2*, and *HOXD12* were up-regulated in MEGAL compared with HSPC or MK progenitors (Fig. 1B). Based on the differentially expressed genes in MEGAL compared with HSPC and MK, we identified one cluster of mutually inclusive up-regulated genes (*n* = 1933) (Fig. 1D) and one cluster for mutually inclusive down-regulated genes (*n* = 2394) (Fig. 1E and Table S3). The direction of changes in gene expression between MEGAL and HSPC or between MEGAL and MK showed a high correlation (*r* = 0.92, *P* < 0.01) (Fig. 1F and Table S4). This suggests that SN-214 leads to significant changes in transcriptional programs during leukemic megakaryopoiesis, which are not found during normal megakaryopoiesis. We also examined the expression of specific epigenetic protein-coding genes and found that, similarly to T-ALL cells, *TET2* is significantly down-regulated (*P* < 0.05) in MEGAL cells,^3^ compared with both HSPC (log_2_ fold change (FC) = −7.9) or MK (log_2_ FC = −8.5). DNA methyltransferase 1 (DNMT1) (log_2_ FC = 1) was up-regulated in MEGAL, compared with MK, while DNMT3B was up-regulated in MEGAL compared with both HSPC (log_2_ FC = 2.1) or MK (log_2_ FC = 1.2) (Fig. 1G). We observed that the promoters of DNMT1 and DNMT3B were enriched with H3K4me3, while H3K4me1 and H3K27ac were enriched at the promoters of both genes (Fig. 1H, I). Additionally, we detected the enrichment of CTCF marks at the body of DNMT3B, indicating the potential involvement of an enhancer mechanism. Additionally, we observed an abundance of H3K36me3 in the body, suggesting a possible amplification of these DNMT genes and genome-wide hypermethylation in SN214^+^ leukemia.

**Figure 1:**
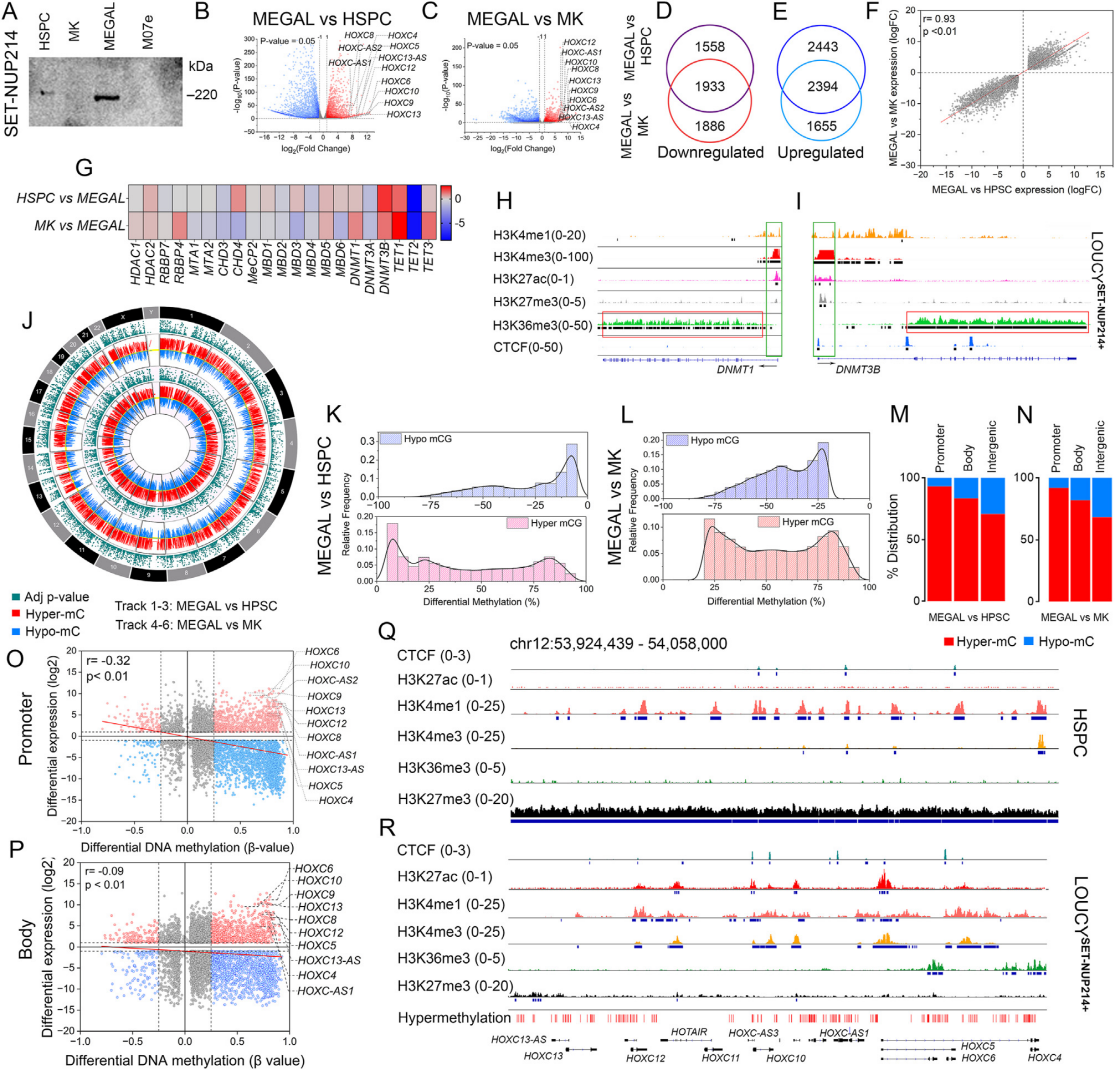
Genome-wide hypermethylation supports enhancer-transcriptional programs in SET-NUP214-expressing leukemia. **(A)** Expression of the SET-NUP214 (SN-214) fusion protein in MEGAL cells was confirmed with Western blot. **(B)** Differential gene expression of genes in MEGAL. A volcano map shows differential expression in MEGAL cells compared with CD34^+^ hematopoietic stem progenitor cells (HSPC), or in **(C)** CD41^+^ normal megakaryocyte progenitor cells (MK). **(D, E)** The mutually inclusive and differentially up-regulated or down-regulated gene cluster in MEGAL compared with HSPC and MK. **(F)** Correlation of differentially expressed genes in MEGAL. A strong correlation (*r* = 0.92) was observed between the differentially expressed genes in MEGAL compared with HSPC and MEGAL compared with MK progenitors. **(G)** Expression of epigenetic protein-coding genes. A heatmap demonstrates the differential expression of selective epigenetic protein-coding genes in MEGAL cells. **(H, I)** Chromatin regulators of DNMT1 and DNMT3B. Enrichment of activating histone marks was observed at the promoter (H3K27ac, H3K4me1, H3K4me3) or body (H3K36me3) of DNMT1 and DNMT3B. **(J)** Genome-wide distribution of hypermethylated and hypomethylated CpG sites in MEGAL compared with HSPC and MK. **(K, L)** Frequency distribution of the magnitude of differentially methylated CpGs in MEGAL compared with HSPC and MK progenitors. **(M, N)** Distribution percentage of hypermethylated and hypomethylated CpGs across the promoters, gene bodies, and intergenic regions in MEGAL compared with HSPC and MK progenitors. Correlation between differential methylation and differential expression of genes at the promoters **(O)** and gene bodies **(P)** in MEGAL compared with HSPC. **(Q, R)** Distribution of DNAm and chromatin marks on HOXC cluster genes. HOXC cluster genes are under the regulation of H3K27me3 in HSPC. In contrast, regulatory transcription factor CTCF and activating chromatin marks (H3K4me1, H3K4me3, H3K27ac, and H3K36me3) are observed on HOXC cluster genes in SN-214^+^ LOUCY cells. Notably, this region coincided with hypermethylated CpGs in MEGAL cells.

A genome-wide profiling of mCs revealed that the majority (>85%) of CpGs across the chromosomes were hypermethylated (mC > 20%) in MEGAL compared with HSPC or MK (Fig. 1J; Fig. S1 and Table S5, 6). The distribution of mCs in terms of magnitude indicates that mC with changes in DNAm of ±20% exhibit the highest relative frequency (Fig. 1K, L). We also observed mC with over 75% increased DNAm in MEGAL, compared with MK, suggesting significant changes in DNAm pattern between normal MK and leukemic MK cells.^4^ A high degree of hypermethylation was also observed at the gene promoters (98% ± 0.68%), gene bodies (81% ± 0.87%), and intergenic regions (84% ± 0.39%) of MEGAL compared with both HSPC and MK (Fig. 1M, N). We observed a good correlation between DNAm and expression in the promoter regions (*r* = −0.32), relative to the gene bodies (*r* ≤ −0.1) in MEGAL, compared with HSPC (Fig. 1O, P) or MK (Fig. S2). Two significant clusters were identified, comprising genes that showed hypermethylation (mC > 25%) and were either down-regulated (*n* = 2099 compared with HSPC, and *n* = 1581 compared with MK) or up-regulated (*n* = 853 compared with HSPC, and *n* = 744 compared with MK) in MEGAL (Table S7, 8). These epigenetically regulated genes were primarily enriched for IL2-STAT5 signaling, epithelial–mesenchymal transition, or up-regulated KRAS signaling among other pathways (Fig. S3 and Table S9, 10). Among the hypermethylated up-regulated genes in MEGAL, we observed ten candidates from the *HOXC* gene cluster (*HOXC4*, *HOXC6*, *HOXC8*, *HOXC9*, *HOXC10*, *HOXC12*, *HOXC13*, *HOXC13-AS*, *HOXC-AS1*, and *HOXC-AS2*). The HOX cluster of genes has previously been reported to promote increased epithelial–mesenchymal transition, cell adhesion, invasion, and metastasis in different cancers.^5^ Therefore, the overexpression of HOXC cluster genes indicates a potential cell adhesion-mediated drug resistance mechanism in SN-214^+^ AML. Finally, we assessed whether these hypermethylated CpGs in SN-214^+^ AML coincided with chromatin regulators, controlling the expression of the target genes, using the HOXC cluster as an example. The genomic region (133 kb) spanning the HOXC cluster genes shows characteristics of bivalent chromatin in HSPC or MK progenitors, with uniformly distributed repressive H3K27me3 marks along with locus-specific enrichment of activating H3K4me1 (Fig. 1Q; Fig. S4). In contrast, the H3K27me3 marks were replaced by activating H3K4me3 and H3K27ac at the promoters or H3K36me3 at the bodies of specific HOXC genes in SN-214^+^ leukemia (Fig. 1R). We also observed an enrichment of CTCF binding at the hypermethylated CpGs in the HOXC gene cluster indicating an SN-214 fusion-induced enhancer-transcriptional mechanism in this disease.

In conclusion, we have demonstrated that SN-214 fusion induces a genome-wide hypermethylation pattern, working cooperatively with regulatory chromatin and transcription factors to activate gene expression, as exemplified with the HOXC cluster genes. No significant changes were observed in the expression of HOXC genes in MK progenitors compared with HSPCs. This suggests that while HOXC genes are not up-regulated during normal megakaryopoiesis, they are up-regulated during leukemic megakaryopoiesis. These epigenetically regulated genes are involved in epithelial–mesenchymal transition or cell adhesion-mediated drug resistance. Our preliminary data also provides a rationale for targeting these genes to treat this rare yet fatal subtype of leukemia.

## Author contributions

S.R.C. has conceptualized and supervised the work and wrote the manuscript. A.K. developed the bioinformatics pipeline and analyzed the methylation and expression data.

## Funding

The study was supported by grants from the National Institutes of Health (No. P20GM121293) to S.R.C. The study was partly funded by the Seeds of Science Award and start-up from the University of Arkansas for Medical Sciences (UAMS) and Arkansas Children's Research Institute (ACRI) to S.R.C.

## Data availability

The arrayed data have been provided as supplementary information. The raw datasets are available from the corresponding author upon reasonable request.

## Conflict of interests

The authors declared no conflict of interests.
