# Supplementary material for: SET-NUP214-induced hypermethylation landscape promotes abnormal overexpression of HOXC cluster genes in acute megakaryoblastic leukemia
Source: Genes Dis. 2024 May 7;12(2):101320. doi: 10.1016/j.gendis.2024.101320 (PMC11615591; doi:10.1016/j.gendis.2024.101320)
Supplement: Multimedia component 1 [file mmc1.docx]

SET-NUP214-induced hypermethylation landscape promotes abnormal overexpression of HOXC cluster genes in acute megakaryoblastic leukemia.

**Supplementary Information**

**Methods**

*Culture and expansion of primary cells and AML cell line.*

Umbilical cord blood-driven CD34+ hematopoietic stem progenitor cells (HSPC) cells (# 70008, STEMCELL Technologies) were cultured in StemSpan SFEM-II media (# 70008, STEMCELL Technologies) supplemented with growth factors SCF (# 300-07, Peprotech), TPO (# 300-18, Peprotech), FLT3L (# 300-19, Peprotech), IL3 (# 200-03, Peprotech), and IL6 (# 200-06, Peprotech) each at a concentration of 10 ng/mL. Additionally, we used UM729 (# 72332, STEMCELL Technologies), a pyrimido-indole derivative, to restrict the lineage differentiation of HSPC. The CD34+ HSPC was the precursor for differentiating the MK progenitors, so HSPC was transferred to SFEM-II media containing a megakaryocyte (MK) expansion supplement (# 02696, STEMCELL Technologies). HSPC were FACS sorted based on CD34+/CD45+, and MK were sorted based on CD41+/ CD45+ surface markers. Sorted cells were used for the extraction of genomic materials. SET-NUP214 fusion-positive MEGAL cell line (ACC719, DSMZ) was cultured in RPMI 1640 media containing 20% FBS, 1% penicillin-streptomycin, and 1% L-glutamine. Cells were periodically checked to nullify the possibilities of mycoplasma contamination.

*Extraction of genomic materials.*

At least 5 X 10^6^ live cells were harvested to extract genomic DNA (gDNA) and RNA using the All-Prep DNA/RNA Mini Kit (# 80204, QIAGEN). The concentration of extracted genomic materials was checked with a qubit spectrometer against the broad-range dsDNA (# Q32850, Thermo Scientific) or RNA (# Q10210, Thermo Scientific) assay kit, respectively. The integrity of the extracted genomic materials was determined with the 4200 Tapestation System (# G2991BA, Agilent Technologies).

*RNA sequencing and analysis.*

To prepare libraries, 100 ng of purified and quality-checked RNA samples (n=3) were submitted to Novogene. Libraries were sequenced using 150 bp paired-end reads on the Illumina NovaSeq 6000 Sequencing System. RNA-seq data was analyzed using the transcript aligner STAR (v2.5.1b) ^1^, and transcript level data were generated by Salmon (v0.7.2)^2^. Differential expression analyses were performed for genes between MEGAL and HSPC, or MEGAL and MK, considering 2-fold changes (FC, p <0.05, log2 scale) in expression as the significant changes.

*Methylation EPIC 935K array.*

The raw iDAT files for 935K data were imported and processed using minfi R package ^3^. The data were normalized using Subset-quantile Within Array Normalization (SWAN) ^4^. SWAN method is known to reduce the technical variability between the probe types while maintaining the biological differences. Methylation levels for each probe are recorded as beta(β) values, which represent the proportion of methylated(M) over methylated plus unmethylated (U) probes and a constant c = 100 ($\beta= M/{(M+U+c)})$. 101532 probes with β-value > 0.8 across all samples and 86157 probes with β-value < 0.2 across all samples were excluded. Another, 1658 probes with cross-hybridization, high detection p-value (> 0.01) and near single-nucleotide polymorphisms were also excluded. The pre-processed and filtered DNAm β-value values were logit transformed to M values approximated as ${log}_{2}[\beta/{(1-\beta)]}$, to ensure a better fit to statistical model assumptions. R-package limma ^5^ was used to assess the statistical significance of difference in methylation values across groups. Probes with absolute percentage difference in β-value > 5% and FDR p-value < 0.05 were considered significant. Significant probes were then mapped to gene symbol using the illumina annotation for EPIC-v2 arrays (EPIC-8v2-0_A1). The methylation levels at significant probes were integrated with gene expression data based on GENCODE version 41(GRCh38.p13) transcript ID. Probes in regions 1.5 kb upstream and 200 bp downstream from the TSS were marked as promoter region probes, while the ones in region immediately downstream of the defined promoters to the 3` ends were marked as gene body probes. Probes in the remaining regions were classified as intergenic probes.

*Intersection and correlation analysis for DNAm and expression*

A Venn intersection analysis (Venny 2.1) was performed to detect the mutually inclusive and differentially upregulated or downregulated genes in MEGAL, compared to HSPC or MK. A linear fit model using an ANOVA was used to evaluate the correlation between the differentially expressed genes between MEGAL and HSPC and MEGAL and MK. We further calculated the median DNAm of the CpG sites at the promoter and body of a certain gene. A correlation analysis was performed with the differential median DNAm (β ≥ ±25%, p<0.05, log2) and differential (log2FC> 2, p< 0.05,) expression of genes.

*Integration of DNAm and chromatin immunoprecipitation sequencing (ChIP-seq) data*

We used the available chromatin modifications data from a SET-NUP214 positive T-cell acute lymphoblastic leukemia (T-ALL) cell line LOUCY. We have visualized several chromatin marks that are overlapped with differentially methylated probes, including H3K4me1 (ENCFF949YUL; replicate 1 and 2), H3K4me3 (ENCFF638PLM; replicate 1 and 2), H3K36me3 (ENCFF546VMH; replicate 1 and 2), H3K27me3 (ENCFF055MHI; replicate 1 and 2) and CTCF (ENCFF125UYZ; replicate 1 and 2) as deposited from Bradley Bernstein group at the Broad Institute. The chromatin marks including H3K4me1 (ENCFF657HBK; replicate 1 and 2), H3K4me3 (ENCFF795ZQG; replicate 1 and 2), H3K36me3 (ENCFF772FHW; replicate 1 and 2), H3K27me3 (ENCFF949ZLD; replicate 1 and 2) marks from CD34+ HSPC were used by using the data, deposited from Bradley Bernstein group at the Broad Institute. The CTCF binding data in CD34+ HSPC was used from the GEO (GSM3762802) dataset as deposited by Young Cheng group at the St. Jude Childrens Research Hospital. The chromatin marks including H3K4me1 (ENCFF925CDE; replicate 1 and 2), H3K4me3 (ENCFF778YJF; replicate 1 and 2), H3K36me3 (ENCFF752NBL; replicate 1 and 2), H3K27me3 (ENCFF407HLL; replicate 1 and 2) marks from CD41+ murine MK progenitors were used by using the data, deposited from Ross Hardison group at the Penn State University. Mapping and coincidence of differentially methylated CpGs (.bed file) and chromatin marks (. bigwig files) were visualized with UCSC genome browser.

*Pathway enrichment analysis*

We have used ShinyGO (v 0.77), a graphical gene-set enrichment analysis (GSEA) for the differentially methylated and expressed genes^6^. The prediction of pathways were performed with hallmark, MSigDB, and have been tabulated in Table S9 and S10. The top 10 pathways have been further plotted based on their enrichment score and false discovery rate (FDR).

*Statistical analysis and software*

A two-way ANOVA was used to determine the significance in differential DNAm analysis. A non-parametric two tailed Mann-Whitney U test was performed for rest of the analyses and statistical analyses were determined at a p value < 0.001 or < 0.05 (as indicated). We used GraphPad Prism (v 7.0) or Origin Pro (v 2023b) software to prepare the graphical plots. The circus plot demonstrating distribution of DNAm was created with Circa (<http://omgenomics.com/circa>).


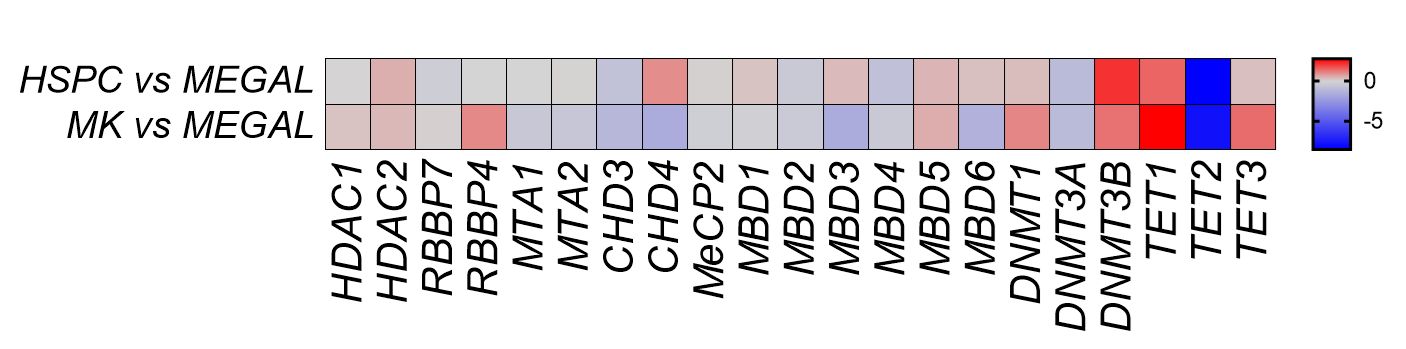
**Results**

**Fig. S1**. A heatmap demonstrating the differential expression of selective epigenetic protein coding genes in SET-NUP214 fusion positive MEGAL cell line, compared to HSPC or MK. We observed significant (log2FC> 2, p<0.05) upregulation of *DNMT3B* (FC=2.1, 5.85E-73) in MEGAL, compared to HSPC. In contrast, *TET2* was dramatically downregulated in MEGAL compared to both HSPC (FC= -8.53047, 2.864E-132) or MK (-7.91864, 1.24E-49).

**
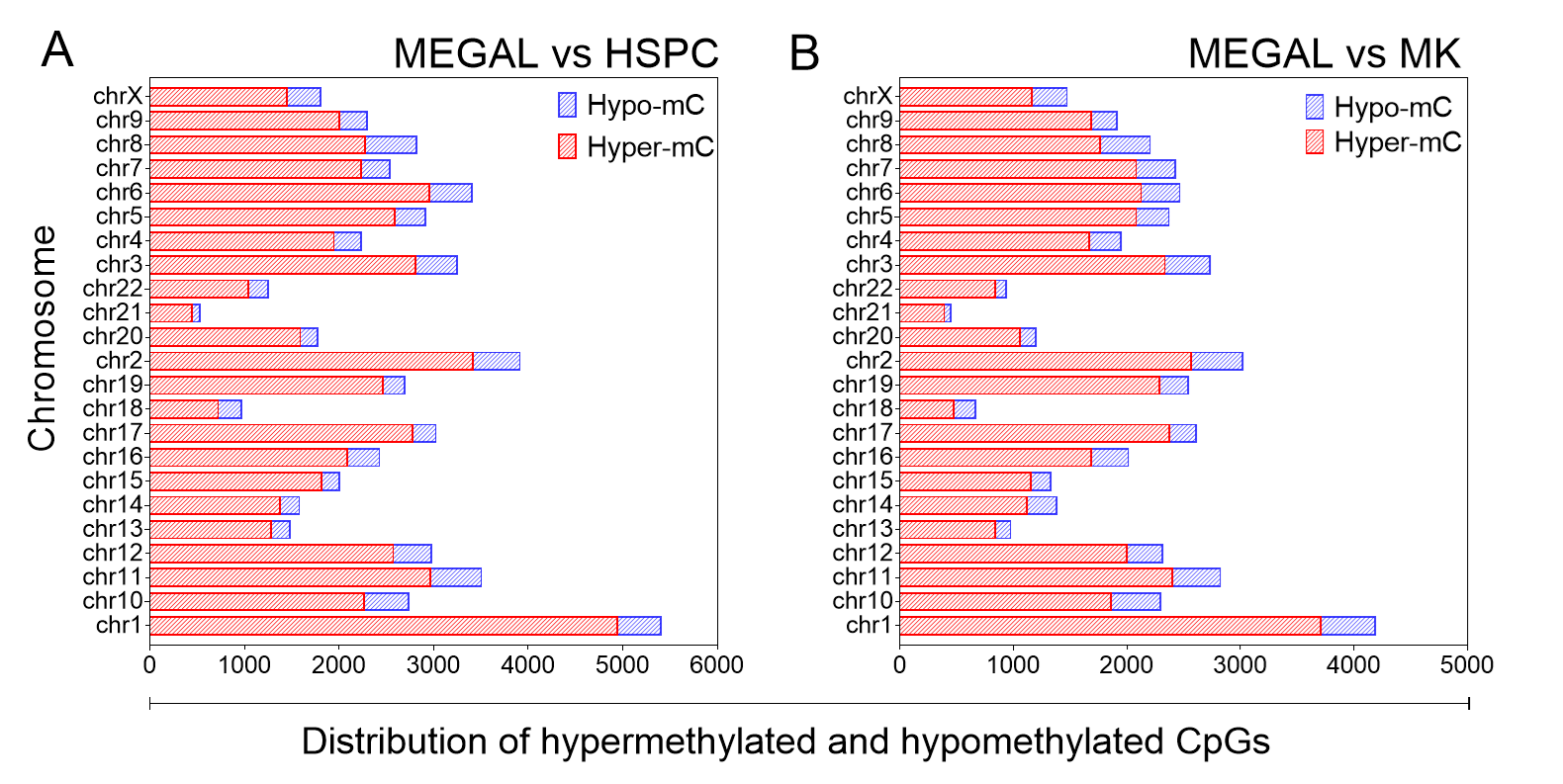
**

**Fig. S2**. Stacked bar plots representing the distribution (count) of hypermethylated (hyper-mCG) and hypomethylated (hypo-mCG) CpG sites across the chromosomes in MEGAL cells compared to HSPC (A) and MK (B).


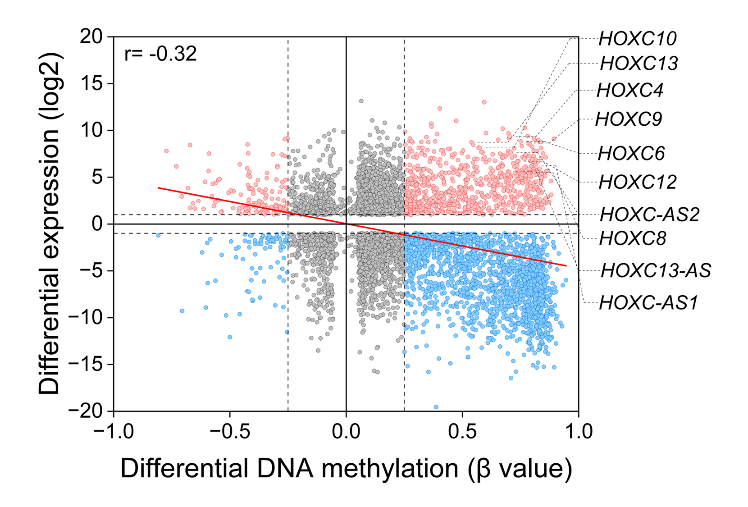

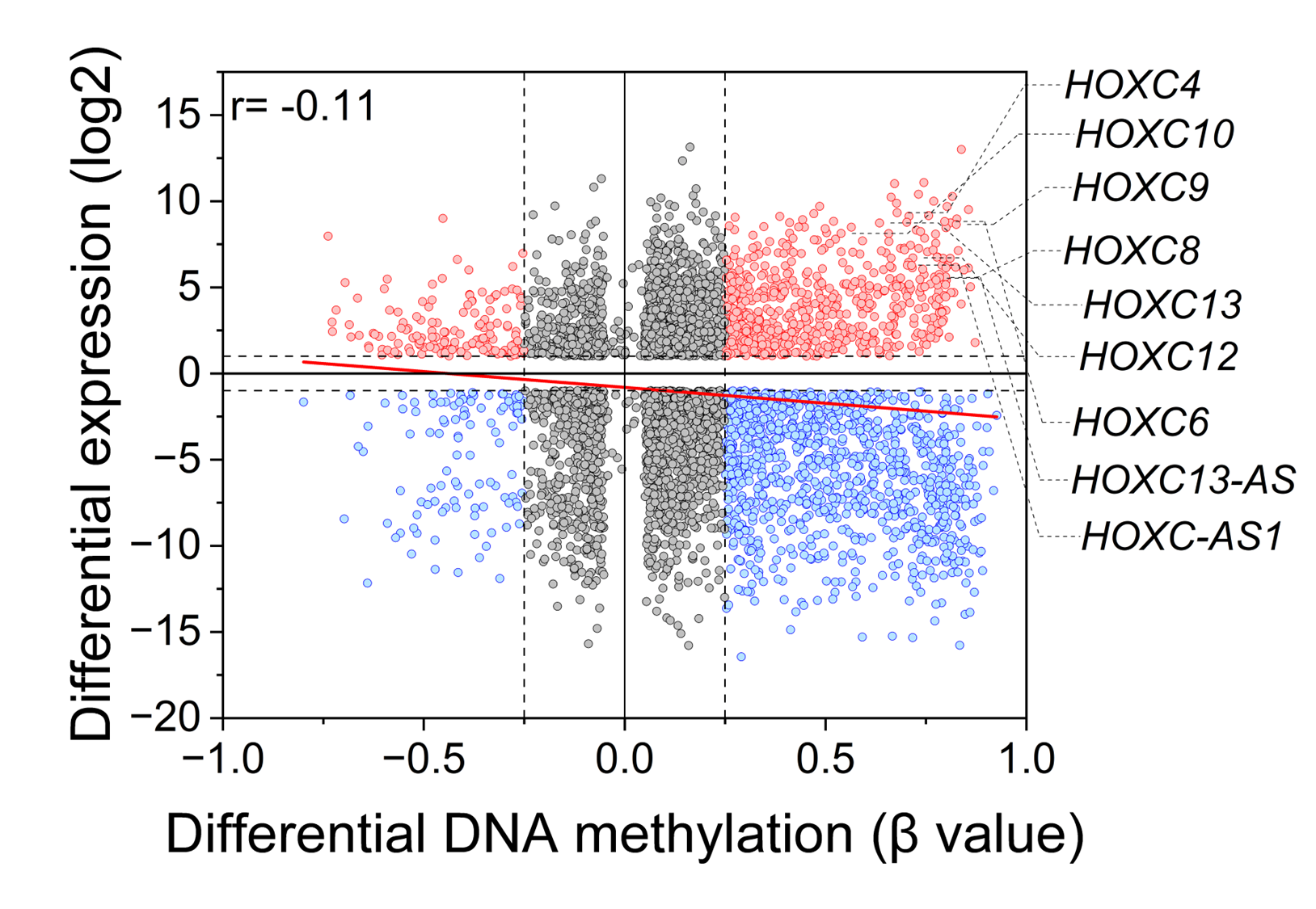


a

b

**Fig. S3**. Correlation analysis with differentially methylated genes and their corresponding expression in the promoter regions (a) and gene bodies (b) in MEGAL, compared to the megakaryocyte progenitors.


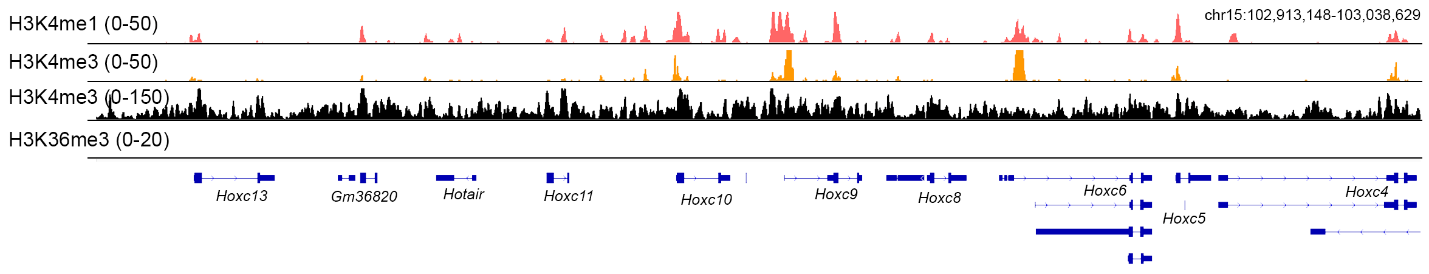


**Fig. S4**. Chromatin immunoprecipitation sequencing demonstrating the distribution of H3K4me1, H3K4me3, H3K36me3, and H3K27me3 across the HOXC cluster genes, as obtained from normal CD41+ murine megakaryocytes.

**References**

1 Dobin, A. *et al.* STAR: ultrafast universal RNA-seq aligner. *Bioinformatics* **29**, 15-21 (2012). <https://doi.org:10.1093/bioinformatics/bts635>

2 Patro, R., Duggal, G. & Kingsford, C. Salmon: accurate, versatile and ultrafast quantification from RNA-seq data using lightweight-alignment. *BioRxiv* **10**, 021592 (2015).

3 Aryee, M. J. *et al.* Minfi: a flexible and comprehensive Bioconductor package for the analysis of Infinium DNA methylation microarrays. *Bioinformatics* **30**, 1363-1369 (2014). <https://doi.org:10.1093/bioinformatics/btu049>

4 Maksimovic, J., Gordon, L. & Oshlack, A. SWAN: Subset-quantile within array normalization for illumina infinium HumanMethylation450 BeadChips. *Genome Biol* **13**, R44 (2012). <https://doi.org:10.1186/gb-2012-13-6-r44>

5 Smyth, G. K. in *Bioinformatics and Computational Biology Solutions Using R and Bioconductor* (eds Robert Gentleman *et al.*) 397-420 (Springer New York, 2005).

6 Ge, S. X., Jung, D. & Yao, R. ShinyGO: a graphical gene-set enrichment tool for animals and plants. *Bioinformatics* **36**, 2628-2629 (2020). <https://doi.org:10.1093/bioinformatics/btz931>
